# Тиреотоксикоз у пациентки с синдромом Шерешевского–Тернера: терапия радиоактивным йодом

**DOI:** 10.14341/probl13132

**Published:** 2022-12-20

**Authors:** Р. М. Гусейнова, А. А. Просвирнина, М. О. Корчагина, А. А. Трухин, М. С. Шеремета

**Affiliations:** Национальный медицинский исследовательский центр эндокринологии; Национальный медицинский исследовательский центр эндокринологии; Первый Московский государственный медицинский университет им. И.М. Сеченова (Сеченовский Университет); Национальный медицинский исследовательский центр эндокринологии; Национальный медицинский исследовательский центр эндокринологии

**Keywords:** синдром Шерешевского–Тернера, аутоиммунитет, тиреотоксикоз, болезнь Грейвса, радиойодтерапия, дозиметрическое планирование, химическое и лекарственно-индуцированное поражение печени

## Abstract

Синдромом Шерешевского–Тернера (СШТ) - хромосомное заболевание, при котором в клетках женского организма одна Х-хромосома нормальная, а другая отсутствует или структурно изменена. Данные генетические аберрации приводят к появлению ряда отклонений в росте и развитии и повышают риск развития аутоиммунных заболеваний, в том числе затрагивающих щитовидную железу (ЩЖ). Патология ЩЖ при СШТ может быть представлена аутоиммунным тиреоидитом (АИТ), гипотиреозом или тиреотоксикозом различного генеза (болезнь Грейвса (БГ), АИТ в стадии тиреотоксикоза).Тиреотоксикоз — синдром, обусловленный избытком циркулирующих в крови гормонов ЩЖ. Одна из основных причин тиреотоксикоза — БГ, органоспецифичное аутоиммунное заболевание, обусловленное выработкой стимулирующих антител к рецепторам тиреотропного гормона. Существует три варианта лечения тиреотоксикоза при БГ: консервативный, хирургический, радионуклидный. Персонализированный подход к лечению особенно важен при наличии у пациента сопутствующих фоновых заболеваний, затрагивающих генотип.Мы представляем клинический случай пациентки Б. с СШТ и БГ, которая направлена к радиологу в отделение радионуклидной терапии ФГБУ «НМИЦ эндокринологии» Минздрава России. Особенности анамнеза: на этапе неонатального скрининга у пациентки диагностирован врожденный гипотиреоз, в возрасте 3 лет инициирована терапия тиреоидными гормонами. В возрасте 21 года у пациентки манифестировал тиреотоксикоз, причиной которого была БГ. На фоне тиреостатической терапии развился токсический гепатит. С учетом непереносимости консервативной терапии рекомендована терапия радиоактивным йодом 131I, в результате которой развился гипотиреоз.

## Abstract (en)

Turner syndrome (TS) is a chromosomal disorder affecting female and characterized by complete or partial monosomy of the X chromosome. These genetic changes lead to the abnormalities in growth and development and increase the risk of autoimmune diseases, including those affecting the thyroid. Thyroid pathology in TS may include autoimmune thyroiditis, hypothyroidism, thyrotoxicosis (Graves disease, AIT in the hyperthyroid state).Thyrotoxicosis is the clinical syndrome of excess circulating thyroid hormones. One of the main causes of thyrotoxicosis is Graves’ disease (GD), an organ-specific autoimmune disease caused by the production of stimulating thyrotropin receptor antibodies. There are three treatment options for thyrotoxicosis: anti-thyroid drugs, radioactive iodine and thyroidectomy. A personalized approach to disease management is especially important in cases of genetic diseases.We present a clinical case of a patient with TS and GD, who has been referred to a radiologist at the Department of Radionuclide Therapy of Endocrinology Research Center. The patient was diagnosed with congenital hypothyroidism at neonatal screening, but thyroid hormones therapy was initiated aged three. Based on the survey, GD was diagnosed aged twenty one. Anti-thyroid drug therapy was started, which resulted in toxic hepatitis. Taking into account intolerance to anti-thyroid drugs, radioiodine therapy has been recommended, which led to hypothyroidism.

## АКТУАЛЬНОСТЬ

Связь между заболеваниями щитовидной железы (ЩЖ) и синдромом Шерешевского–Тернера (СШТ) впервые была предложена Atria и соавт. в 1948 г., когда было сообщено о посмертном обнаружении небольшой ЩЖ с лимфоцитарной инфильтрациейу молодой женщины с СШТ [1]. Известно, что у пациентов с СШТ нередко выявляются аутоиммунные заболевания, в том числе затрагивающие эндокринную систему [2]. Патология ЩЖ включает гипотиреоз, тиреотоксикоз и аутоиммунный тиреоидит (АИТ). АИТ встречается наиболее часто, он может иметь тиреотоксическую фазу или переходить в стойкий гипотиреоз [3–7]. Также возможно носительство антител к тиреопероксидазе без нарушений функции ЩЖ.

Изучая распространенность и частоту заболеваний ЩЖ у взрослых с СШТ, M. El-Mansoury и соавт. обнаружили, что гипотиреоз развивается значительно чаще у лиц с СШТ, чем в контрольной группе (25% против 2%; P<0,0001). При этом нарушение функции ЩЖ с развитием гипотиреоза проявляется довольно рано, в возрасте около 8 лет, чаще у девочек с изохромосомой iXq, что требует тщательного обследования этой когорты пациентов с раннего возраста [8]. АИТ при СШТ имеет тенденцию к переходу в манифестный гипотиреоз или тиреотоксикоз [9].

СШТ — хромосомное заболевание, встречающееся с частотой 1:2000–1:2500 новорожденных девочек [10][11]. В норме в соматических клетках женского организма 46 хромосом, из них две половые Х-хромосомы (кариотип 46, XX), каждая из которых имеет короткое плечо p и длинное плечо q [12]. На ранних этапах эмбрионального развития в каждой клетке женщины одна Х-хромосома случайным образом инактивируется, образуя тельце Барра (половой хроматин) [13]. Данный процесс называется дозовой компенсацией Х-сцепленных генов. В последующем все клетки из одной клеточной линии будут иметь одну и ту же инактивированную Х-хромосому. Таким образом, в части клеток активна Х-хромосома, унаследованная от матери, в других — унаследованная от отца. При СШТ отсутствует вся или часть второй Х-хромосомы, а в результате потери генетического материала возникают нарушения роста и развития. Классический СШТ, моносомия Х-хромосомы (45, X0), возникает из-за нарушения расхождения хромосом в процессе мейоза с утратой отцовской Х-хромосомы и встречается приблизительно в половине случаев. В большинстве из них эмбрион с кариотипом 45, X0 не выживает после I триместра, 99% беременностей прерываются спонтанно. Около 10% спонтанных абортов в I триместре беременности связаны с моносомией Х [14][15]. Также существует мозаичный СШТ, при котором клетки различаются по хромосомному набору [16]. В некоторых случаях присутствуют аномалии в Х-хромосоме или есть Y-хромосома [3][11]. Данные о влиянии кариотипа СШТ на распространенность таких аутоиммунных заболеваний, как АИТ, целиакия, сахарный диабет 1 типа, псориаз, витилиго, гнездная алопеция, воспалительные заболевания кишечника, противоречивы [5]. По одним из них, наличие изохромосомы iXq повышает риск аутоиммунного поражения ЩЖ. Другие исследователи не обнаружили связи между аутоиммунной патологией ЩЖ и различными кариотипами СШТ [3][5][17][18]. Более высокая распространенность болезни Грейвса (БГ) у пациентов с СШТ по сравнению с общей популяцией остается под вопросом [5][19].

Распространенность аутоиммунных заболеваний при СШТ увеличивается с возрастом, а также с началом гормональной терапии эстрогенами [20][21]. Примерно у 40–60% пациентов с СШТ выявляются аутоантитела к ЩЖ, однако какие-либо клинические симптомы наблюдаются редко [3]. По данным M. Wegiel и соавт., у 14,9% пациентов с СШТ развивается АИТ без каких-либо клинических проявлений гипотиреоза [5]. Несмотря на более высокий риск аутоиммунных заболеваний у пациентов с СШТ по сравнению с общей популяцией, ассоциация с БГ встречается редко, всего в 1,7–3,0% случаев [19][22–24]. По данным Т. Aversa и соавт., у большинства пациентов (67,7%) с СШТ субклинический гипотиреоз на фоне АИТ через ~5 лет переходит в манифестный [24]. Было также отмечено, что пациенты с СШТ имеют более высокий риск развития БГ, чем в общей популяции, что может объясняться аутоиммунитетом [23–25]. По данным S. Mohamed, общая распространенность аутоиммунных заболеваний ЩЖ у пациенток с СШТ составила 38,6% (95% доверительный интервал — ДИ 29,7–47,6%), из них 12,7% (95% ДИ 9,30–16,1%) имели манифестный гипотиреоз и 2,6% (95% ДИ 1,5–3,8%) — тиреотоксикоз [26].

Ведение пациентов с СШТ и БГ — сложная задача в медицинской практике. Как правило, такие пациенты получают консервативную терапию тиреостатиками (ТС) [23][27]. Непереносимость, плохая переносимость ТС или их неэффективность становятся причиной выбора другого метода лечения (рис. 1). При анализе литературы мы не обнаружили клинических случаев, описывающих использование радиойодтерапии (РЙТ) для лечения БГ у пациентов с СШТ.

Мы представляем клинический случай пациентки с СШТ и БГ, находившейся на лечении в отделении радионуклидной терапии ФГБУ «НМИЦ эндокринологии» Минздрава России (НМИЦ эндокринологии).

**Figure:**
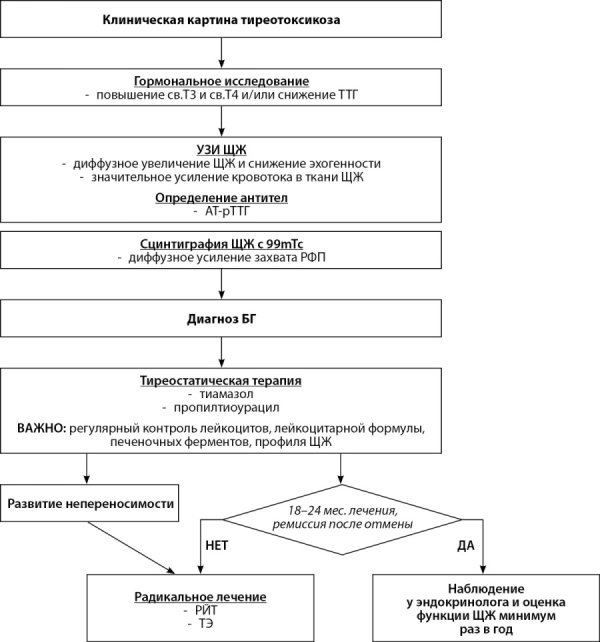
Рисунок 1. Алгоритм ведения пациента: от диагностики до лечения и наблюдения (адаптированный вариант) [28].Figure 1. Patient management algorithm: from diagnosis to treatment and observation (adapted version) [28].

## ОПИСАНИЕ СЛУЧАЯ

Пациентка Б., 21 год, обратилась 22.11.2021 в отделение радионуклидной терапии НМИЦ эндокринологии с диагнозом: «Болезнь Грейвса. Субклинический тиреотоксикоз» для решения вопроса о проведении РЙТ. РЙТ была рекомендована в связи с непереносимостью ТС в виде токсического гепатита, рисками хирургического лечения и наличием сопутствующей генетической патологии.

Наблюдается у гинеколога-эндокринолога с диагнозом: «СШТ, врожденный гипотиреоз». По данным цитогенетического исследования в НМИЦ гематологии в 2019 г. выявлен кариотип с потерей Х-хромосомы (45, Х0). На момент поступления состояние удовлетворительное, сознание ясное. Рост 154 см, масса тела 29,9 кг, ИМТ 12,6 м2. Пациентка получает терапию левотироксином и эстроген-гестагенными препаратами (Эстрожель гель 0,5 г, Дюфастон 200 мкг/сут).

Врожденный гипотиреоз диагностирован в ходе неонатального скрининга. Терапия левотироксином инициирована с 3-летнего возраста в дозе 25 мкг (уровень ТТГ неизвестен), с последующей титрацией до 56 мкг в сутки и отменой препарата в возрасте 21 года, в августе 2021 г. в связи со снижением уровня ТТГ, ухудшением самочувствия (слабость, тахикардия, тремор рук, резкое снижение массы тела). При дообследовании установлен манифестный тиреотоксикоз (табл. 1).

**Table:** Таблица 1. Тиреоидный профиль, активная стадия заболевания (август 2021 г.)Table 1. Thyroid profile, active disease (August 2021) Примечание. ТТГ — тиреотропный гормон; св.Т4 — свободный тироксин; св.Т3 — свободный трийодтиронин; АТ к ТПО — антитела к тиреопероксидазе.

Показатель	Значение	Референсный интервал
ТТГ, мЕд/л	0,004	0,4–4,6
св.Т4, пмоль/л	36,9	11–22
св.Т3, пмоль/л	9,78	3,8–7,3
АТ к ТПО, МЕ/мл	600	0–34

Проведено обследование у офтальмолога — данных за наличие эндокринной офтальмопатии не обнаружено.

Назначена терапия тиамазолом в дозе 30 мг с последующей титрацией до 15 мг в сутки. Стоит отметить, что терапия ТС была инициирована в поликлинике по месту жительства без предварительного уточнения генеза тиреотоксикоза. На фоне приема препарата в течение 1 мес пациентка отметила иктеричность склер, по данным биохимического анализа крови выявлено повышение печеночных трансаминаз (табл. 2). Проведена дифференциальная диагностика заболеваний печени: не было никаких данных, подтверждающих аутоиммунный гепатит, первичный склерозирующий холангит или первичный билиарный цирроз. Не было выявлено метаболических заболеваний печени, связанных с дефицитом альфа-1-антитрипсина, болезнью Вильсона–Коновалова, гемохроматозом, а также вирусных гепатитов. Доброкачественные образования печени также исключены. Отсутствие холедохоэктазии опровергало первичное поражение желчевыводящей системы. Пациентка переведена натерапию пропилтиоурацилом 50 мг в сутки. Так как на фоне терапии пропилтиоурацилом сохранялись субклинический тиреотоксикоз и повышение печеночных трансаминаз, препарат был отменен.

**Table:** Таблица 2. Лабораторные показатели пациентки Б. на фоне тиреостатической терапии от октября 2021 г.Table 2. Laboratory parameters of patient B. against the background of thyreostatic therapy from October 2021 Примечание. ТТГ — тиреотропный гормон; св.Т4 — свободный тироксин; св.Т3 — свободный трийодтиронин; ЩФ — щелочная фосфатаза; АЛТ — аланинаминотрансфераза; АСТ — аспартатаминотрансфераза; ГГТП — гамма-глутамилтрансфераза.

Показатель на фоне приема тирозола 30–15 мг/сут	Значение	Референсный интервал
ТТГ, мМЕ/л	0,173	0,4–4,6
св.Т3, пмоль/л	4,58	3,8–7,3
св.Т4, пмоль/л	16,69	11,0–22,0
ЩФ, Ед/л	303	38–118
АЛТ, Ед/л	67	5–40
АСТ, Ед/л	55,9	5–40
ГГТП, Ед/л	134	5–40
На фоне приема пропилтиоурацила 50 мг/сут		
ТТГ, мМЕ/л	0,028	0,4–4,6
св.Т3, пмоль/л	8,06	3,8–7,3
св.Т4, пмоль/л	26,9	11,0–22,0
ЩФ, Ед/л	366	38–118
АЛТ, Ед/л	78	5–40
АСТ, Ед/л	67	5–40
ГГТП, Ед/л	113	5–40
билирубин общий, мкмоль/л	27,4	0–20,5
билирубин прямой, мкмоль/л	19,2	0–9

По данным ультразвукового исследования (УЗИ) ЩЖ в ноябре 2021 г. были выявлены признаки диффузного токсического зоба. Общий объем ЩЖ составил 24,5 см3, было отмечено неравномерное снижение эхогенности и усиление васкуляризации ЩЖ. Наряду с сохраняющимся субклиническим тиреотоксикозом впервые обнаружены антитела к рецепторам тиреотропного гормона (АТ-рТТГ), что позволило подтвердить диагноз БГ (табл. 3).

**Table:** Таблица 3. Динамика лабораторных показателей от ноября 2021 г.Table 3. Dynamics of laboratory parameters from November 2021 Примечание. ТТГ — тиреотропный гормон; св.Т4 — свободный тироксин; св.Т3 — свободный трийодтиронин; АТ-рТТГ — антитела к рецепторам тиреотропного гормона.

Показатель	Значение	Референсный интервал
ТТГ, мЕд/л	0,033	0,4–4,6
св.Т4, пмоль/л	15,4	11–22
св.Т3, пмоль/л	4,96	3,8–7,3
АТ-рТТГ, МЕ/л	8,96	0–1,75

21 ноября 2021 г. пациентка проконсультирована радиологом НМИЦ эндокринологии, методом выбора радикального лечения стала терапия радиоактивным йодом (131I). На диагностическом этапе выполнена сцинтиграфия ЩЖ с 99mTc-пертехнетатом, по данным которой отмечены признаки повышенного захвата радиофармпрепарата (РФП) диффузного характера в обеих долях ЩЖ, повышение общего индекса захвата 99mTc-пертехнетата — 10,3% при норме от 0,7 до 1,8% (рис. 2).

**Figure:**
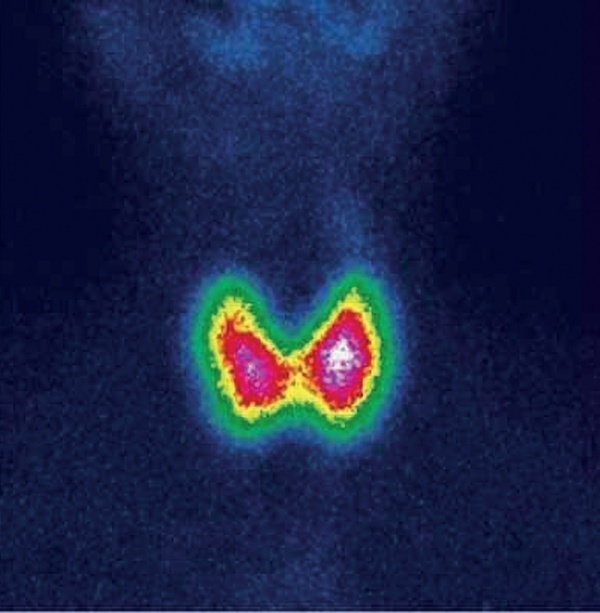
Рисунок 2. Сцинтиграфия ЩЖ с 99mТс-пертехнетатом после внутривенного введения РФП (29.11.2021). Признаки диффузного усиления захвата РФП.Figure 2. Thyroid scintigraphy with 99mTc-pertechnetate after intravenous administration of radiopharmaceuticals (11/29/2021). Signs of diffuse enhancement of radiopharmaceutical capture.

Для расчета эффективной и безопасной терапевтической активности 131I было выполнено индивидуальное дозиметрическое планирование. 30.11.2021 г. введена трейсерная активность 131I в размере 9,2 МБк. Средневзвешенный объем долей ЩЖ поУЗИ и сцинтиграфии для левой доли составил 10,9 мл, правой — 9,7 мл, распределение 131I в левой доле равно 52%, в правой — 48%, индекс накопления 131I на 24 ч составил 28% на обе доли ЩЖ (рис. 3).

**Figure:**
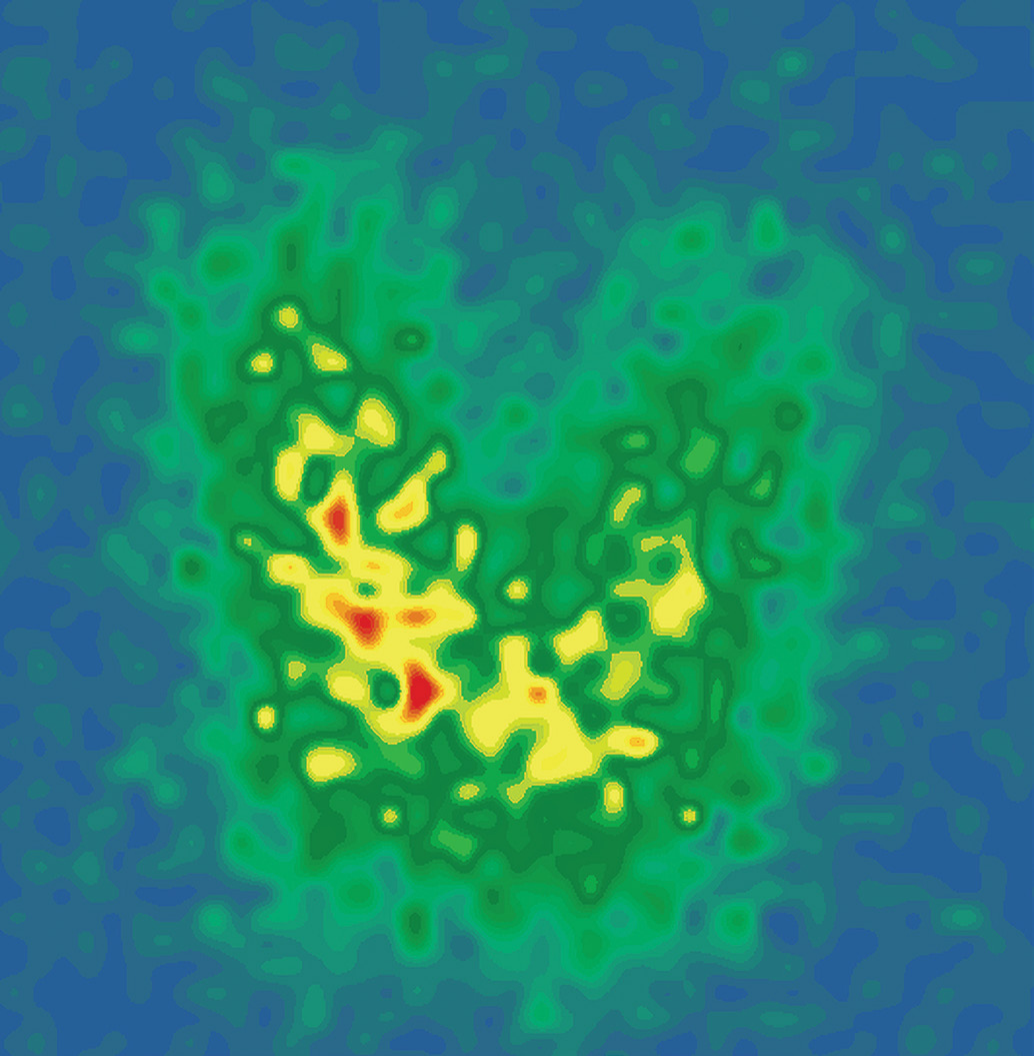


Полученные результаты дозиметрического планирования указывали на прогнозируемое снижение терапевтического эффекта 131I, поэтому пациентке рекомендовано динамическое наблюдение у эндокринолога без дополнительной медикаментозной терапии. При наблюдении в динамике, через неделю, по данным лабораторного обследования, зафиксированы многократное повышение активности печеночных трансаминаз и снижение функции ЩЖ, инициирован прием левотироксина в дозе 25 мкг/сут с последующей титрацией до 56 мкг/сут. Через 4 нед на фоне приема левотироксина развился тиреотоксикоз (табл. 4).

Таблица 4. Динамика лабораторных показателей после введения 131I и на фоне терапии левотироксином.Table 4. Dynamics of laboratory parameters after administration of 131I and during therapy with levothyroxine. Дата исследования Показатель Значение Референсный интервал 07.12.21 После введения 131I ТТГ, мМЕ/л св.Т4, пмоль/л св.Т3, пмоль/л АТ к рТТГ, МЕ/л ЩФ, Ед/л ГГТП, Ед/л АСТ, Ед/л АЛТ, Ед/л 9,002 12,0 3,37 11,31 614 140 265 386 0,4–4,6 11–22 3,8–7,3 0–1,5 70–290 0–20 5–40 5–40 15.12.21 ТТГ, мМЕ/л св.Т4, пмоль/л св.Т3, пмоль/л ЩФ, Ед/л ГГТП, Ед/л АСТ, Ед/л АЛТ, Ед/л 33,6 6,77 2,7 226 92 34,4 59,3 0,4–4,6 12–20 3,6–6,7 30–120 38 35 35 29.12.21 на фоне терапии левотироксином 56 мкг/сут ТТГ, мМЕ/л св.Т3, пмоль/л АТ-рТТГ, Ед/л 0,027 12,8 8,5 0,4–4,6 3,6–6,7 0–1,5 Примечание. ТТГ — тиреотропный гормон; св.Т4 — свободный тироксин; св.Т3 — свободный трийодтиронин; ЩФ — щелочная фосфатаза; АЛТ — аланинаминотрансфераза; АСТ — аспартатаминотрансфераза; ГГТП — гамма-глутамилтрансфераза. Доза левотироксина снижена до 25 мкг/сут. Проведена коррекция заместительной терапии эстроген-гестагенными препаратами — Эстрожель гель 1 доза аппликатора в сутки в течение 25 дней, Дюфастон 10 мг по 2 табл. в сутки с 14-го по 26-й дни цикла. При дообследовании в январе 2022 г. отмечалась тенденция к нормализации печеночных ферментов. По данным УЗИ ЩЖ от марта 2022 г.: общий объем 23 мл, диффузные изменения ЩЖ, узловых образований не обнаружено. По результатам лабораторного обследования вновь зафиксирован тиреотоксикоз (табл. 5). Таблица 5. Посттерапевтические показатели печеночных ферментов и тиреоидного профиляTable 5. Post-treatment parameters of liver enzymes and thyroid profile Дата исследования Показатель Значение Референсный интервал Январь 2022 АЛТ, Ед/л АСТ, Ед/л ЩФ, Ед/л ГГТП, Ед/л 45,5 54,7 138 78 5–40 5–40 70–290 0–20 Март 2022 ТТГ, мкМЕ/мл св.Т4, нг/дл св.Т3, пг/мл 0 2,39 7,83 0,3–5,5 0,89–1,76 2,3–4,2 Примечание. ТТГ — тиреотропный гормон; св.Т4 — свободный тироксин; св.Т3 — свободный трийодтиронин; ЩФ — щелочная фосфатаза; АЛТ — аланинаминотрансфераза; АСТ — аспартатаминотрансфераза; ГГТП — гамма-глутамилтрансфераза. Учитывая неэффективность и непереносимость ТС, сопутствующую патологию, объем ЩЖ, риски хирургического лечения, рекомендовано проведение радикального лечения путем терапии 131I. При повторном дозиметрическом расчете терапевтической активности (31.03.2022) введена трейсерная активность 131I в размере 7,2 МБк. Средневзвешенный объем долей ЩЖ по УЗИ и сцинтиграфии для левой доли составил 9,51 мл, для правой — 8,18 мл, распределение 131I в левой доле — 52%, в правой доле — 48%, индекс накопления 131I на 24 ч — 42% на обе доли ЩЖ (рис. 4). strong>Рисунок 4. Сцинтиграфия щитовидной железы с 131I на 24 ч после перорального введения трейсерной активности (01.04.2022).Figure 4. Thyroid scintigraphy with 131I at 24 h after oral administration of tracer activity (04/01/2022). Полученные результаты дозиметрического планирования указывали на достаточный для достижения терапевтического эффекта уровень захвата 131I в ткани ЩЖ, поэтому пациентке рекомендована терапевтическая активность в диапазоне от 530 до566 МБк. После РЙТ пациентка наблюдалась в течение 2 мес, нормализация тиреоидного статуса на фоне лечения сопровождалась уменьшением клинических проявлений тиреотоксикоза и положительной динамикой лабораторных показателей функции печени (табл. 6). С 26.04.22 инициирована терапия левотироксином (эутирокс) в дозировке 44 мкг/сут. С 11.05.22 доза левотироксина повышена до 100 мкг/сут. Таблица 6. Динамика лабораторных показателейTable 6. Dynamics of laboratory parameters Дата исследования Показатель Значение Референсный интервал 04.04.2022 ТТГ, мМЕ/мл св.Т4, пмоль/л св.Т3, пмоль/л АТ к рТТГ МЕ/мл АЛТ, Ед/л АСТ, Ед/л 0 2.24 7,57 9,86 30 21 0.4–4.6 11–22 3,8–7,3 0–1,5 5–40 5–40 22.04.2022 ТТГ, мМЕ/мл св.Т4, пмоль/л общ.Т3, пмоль/л 0,71 0,81 0,65 0,35–5,5 0,89–1,76 0,6–1,81 11.05.2022 ТТГ, мМЕ/мл св.Т4, пмоль/л св.Т3, пмоль/л ГГТП, Ед/л ЩФ, Ед/л АЛТ, Ед/л АСТ, Ед/л 45.918 7.04 3.41 93 128 24 33 0.4–4.0 7.7–14.2 3.8–6.8 4–38 30–120 0–35 0–35 Примечание. ТТГ — тиреотропный гормон; св.Т4 — свободный тироксин; св.Т3 — свободный трийодтиронин; ЩФ — щелочная фосфатаза; АЛТ — аланинаминотрансфераза; АСТ — аспартатаминотрансфераза; ГГТП — гамма-глутамилтрансфераза. Пациентке рекомендовано наблюдаться у эндокринолога с целью мониторинга функции ЩЖ и своевременного компенсирования гипотиреоза, а также выявления рецидива тиреотоксикоза (рис. 5). Пациентам с СШТ следует наблюдаться у эндокринолога как минимум 1 раз в 6 мес [3]. Кроме того, учитывая токсический гепатит в анамнезе, рекомендован контроль уровня печеночных ферментов в течение 12–24 мес после проведения РЙТ (первые полгода после РЙТ — 1 раз в 3 мес, далее — 1 раз в полгода). Рисунок 5. Алгоритм проведения РЙТ и посттерапевтического мониторинга пациента.Figure 5. Algorithm for RIT and post-therapy monitoring of the patient. ОБСУЖДЕНИЕ Представленный клинический случай применения радиоактивного йода у пациентки с БГ и СШТ является уникальным. В литературе не описаны случаи перехода врожденного гипотиреоза (ВГ) в тиреотоксикоз. Кроме того, при ВГ терапия тиреоидными гормонами инициируется сразу, поскольку нормализация тиреоидного статуса необходима для обеспечения нормального роста и развития, особенно нервной системы. Назначают левотироксин (начальная доза 10–15 мкг/кг/сут). Непосредственная цель лечения — быстро достичь эутиреоза (повысить уровень тироксина (Т4) и нормализовать уровень тиреотропного гормона (ТТГ)). Частый лабораторный мониторинг в младенческом возрасте необходим для обеспечения оптимального нейрокогнитивного развития. Стоит отметить, что диагноз ВГ не должен ставиться только на основании аномального неонатального скрининга, необходимы подтверждающие тесты (после получения результатов ТТГ за пределами референсных значений, от 9 и более МЕд/л, необходим пересмотр взятого материала, при получении аналогичного результата — забор крови из вены в детской поликлинике на ТТГ и св.Т4) [29][30]. После назначения заместительной терапии сывороточный ТТГ и св.T4 следует измерять через 2 нед и 1,5 мес, далее на первом году жизни 1 раз в 2–3 мес [30]. У пациентки Б. терапия левотироксином инициирована в возрасте 3 лет. При этом отклонений в умственном развитии, характерных для детей с ВГ при несвоевременно начатой терапии левотироксином, у нашей пациентки не выявлено. Это позволяет предположить транзиторный характер ВГ. Его возможные причины: эндемический дефицит йода, пренатальный и постнатальный избыток йода, материнские блокирующие АТ-рТТГ, антитиреоидные препараты, мутация DUOX 2, изолированная гипертиротропинемия (нормальный Т4, высокий ТТГ) [29]. У части новорожденных с транзиторным гипотиреозом в первые месяцы или годы жизни показатели функции ЩЖ могут нормализоваться без назначения терапии тиреоидными гормонами [31]. Мы не знаем, какие показатели ТТГ были у нашей пациентки до назначения левотироксина, но можем предположить, что в течение первых лет жизни был период эутиреоза. Известно, что у родственников первой линии родства нарушений в функции ЩЖ и других эндокринных заболеваний не обнаружено, но данных о состоянии ЩЖ у матери пациентки во время беременности нет. Как уже было отмечено, гипотиреоз при СШТ часто развивается в результате АИТ, а с течением времени конверсия АИТ в БГ возможна при изменении баланса между уровнями стимулирующих и блокирующих АТ-рТТГ [32]. Повышенный риск аутоиммунных заболеваний у пациентов с СШТ связан с недостаточностью Х-хромосомы. Было показано, что гены, расположенные в Х-хромосоме, включая локус основного комплекса гистосовместимости в длинном плече, участвуют в регуляции иммунного ответа и изменении иммунной толерантности [33]. Также аутоиммунная предрасположенность при СШТ обусловлена изменением экспрессии Х-связанного гена FOXP3. Данный ген важен для развития регуляторных Т-клеток, а полная потеря экспрессии FOXP3 приводит к тяжелому аутоиммунитету [34]. Функция ЩЖ при СШТ с возрастом может изменяться, поэтому тиреоидный статус необходимо тщательно контролировать. Тиреотоксикоз при БГ лечится подавлением синтеза тиреоидных гормонов при помощи ТС, полным удалением функционирующей тиреоидной ткани при ТЭ или редукцией объема путем РЙТ. Консервативное лечение тиреотоксикоза может применяться в качестве терапии первой линии или для подготовки к радикальному лечению [35–37]. Частые побочные эффекты ТС (1–5%) — кожный зуд, сыпь и артралгия. К крайне редким, но тяжелым побочным эффектам относятся гепатит, волчаночно-подобный синдром и агранулоцитоз. Считается, что поражение печени при тиреотоксикозе может быть проявлением одного из трех заболеваний: лекарственный гепатит на фоне приема ТС; сопутствующие аутоиммунные заболевания печени; гепатопатии как непосредственное проявление тиреотоксикоза [38–40]. В настоящее время нет четких доказательств прямого токсического действия ТС на печень [41]. По данным литературы, чаще всего отмечается синдром холестаза с повышением уровня показателей гамма-глутамилтранспептидазы и билирубина в сыворотке крови, а также синдром цитолиза с повышением уровней АЛТ и АСТ. Общая частота гепатотоксичности, вызванной любым ТС, составляет <0,5%. При использовании наиболее распространенных ТС, тиамазола и пропилтиоурацила, частота нарушений функции печени, включая небольшое повышение трансаминаз, увеличивается на 15% [42][43]. Выявленные в клиническом примере лабораторные и клинические признаки тиреотоксикоза дали основание к назначению ТС, что привело к гипербилирубинемии и повышению уровня трансаминаз при нормальных предшествующих биохимических показателей крови. Таким образом, токсический гепатит развился на фоне применения ТС, возможность первичного заболевания печени была исключена. Лечение ТС требует регулярного контроля уровня лейкоцитов, печеночных ферментов. Также необходим контроль профиля ЩЖ, поскольку тиреостатическая терапия нередко сопровождается эпизодами гипотиреоза и тиреотоксикоза. Учитывая возможные побочные эффекты длительной тиреостатической терапии, сложность контроля и риск рецидива заболевания после отмены препарата, достигающий 70% и более, все больше специалистов отдают предпочтение радикальным методам лечения [44–46]. В нашем случае тяжесть состояния пациентки, обусловленная развитием токсического гепатита на фоне приема ТС, стала основанием для проведения РЙТ. По некоторым данным, РЙТ может влиять на функцию печени, так как печень активно участвует в метаболизме тиреоглобулина и тиреоидных гормонов, в составе которых есть 131I [47]. В литературе описаны случаи, когда после РЙТ БГ пациенты, у которых ранее дисфункция печени отсутствовала, имели повышение печеночных трансаминаз, а в некоторых случаях наблюдалось лекарственное поражение печени [48]. У трети пациентов, проходящих дозиметрическое планирование при подготовке к РЙТ в НМИЦ эндокринологии, наблюдалась фиксация 131I в области печени от 1,1 до 10,3%, при этом в большинстве случаев фиксация не превышала 4%. Мы предполагаем, что главными предикторами фиксации 131I в тканях печени являются индекс захвата 99mTc, захват 131I на 24 ч и характер удержания 131I в ЩЖ с 24 по 48 ч. При этом невысокой прогностической ценностью обладают такие параметры как объем ЩЖ и дозировка ТС. Эти данные требуют активного изучения. ЗАКЛЮЧЕНИЕ На сегодняшний день патофизиологические аспекты более частого развития аутоиммунной патологии ЩЖ у пациентов с СШТ изучены недостаточно. Предполагается, что она может развиваться в результате гаплоинсуффективности генов Х-хромосомы, избыточной продукции провоспалительных цитокинов, снижения уровня противовоспалительных цитокинов и первичной яичниковой недостаточности. Дальнейшее изучение аутоиммунных нарушений при СШТ может способствовать лучшему пониманию патогенеза аутоиммунных заболеваний в целом. Ведение пациентов с СШТ и БГ должно производиться с привлечением мультидисциплинарной команды специалистов и с учетом современных позиций персонализированной медицины. Существующие на сегодняшний день методы лечения позволяют полностью избавить пациента от тиреотоксикоза и повысить качество его жизни. Проведение РЙТ у пациентов с СШТ или другой хромосомопатией должно рассматриваться индивидуально с учетом возможных рисков по развитию осложнений РЙТ и результатов проведенного предварительного обследования. Важно изучать влияние РЙТ тиреотоксикоза на функцию печени с целью дифференциальной диагностики ее нарушений. ДОПОЛНИТЕЛЬНАЯ ИНФОРМАЦИЯ Источники финансирования. Работа выполнена по инициативе авторов без привлечения финансирования. Конфликт интересов. Авторы декларируют отсутствие явных и потенциальных конфликтов интересов, связанных с содержанием настоящей статьи. Участие авторов. Все авторы внесли одинаковый вклад в написание статьи. Все авторы одобрили финальную версию статьи перед публикацией, выразили согласие нести ответственность за все аспекты работы, подразумевающую надлежащее изучение и решение вопросов, связанных с точностью или добросовестностью любой части работы. Согласие пациента. Пациентка добровольно подписала информированное согласие на публикацию персональной медицинской информации в обезличенной форме в журнале «Проблемы эндокринологии».
